# First-Principle Insights Into Molecular Design for High-Voltage Organic Electrode Materials for Mg Based Batteries

**DOI:** 10.3389/fchem.2020.00083

**Published:** 2020-02-18

**Authors:** Johann Lüder, Sergei Manzhos

**Affiliations:** ^1^Department of Materials and Optoelectronic Science, National Sun Yat-sen University, Kaohsiung City, Taiwan; ^2^Centre Énergie Matériaux Télécommunications, Institut National de la Recherche Scientifique, Varennes, QC, Canada

**Keywords:** magnesium battery, ab initio modeling, organic cathode material, rational design, DFT, molecular design

## Abstract

Low cost, scalability, potentially high energy density, and sustainability make organic magnesium (ion) battery (OMB) technologies a promising alternative to other rechargeable metal-ion battery solutions such as secondary lithium ion batteries (LIB). However, most reported OMB cathode materials have limited performance due to, in particular, low voltages often smaller than 2 V vs. Mg^2+^/Mg and/or low specific capacities compared to other competing battery technologies, e.g., LIB or sodium ion batteries. While the structural diversity of organic compounds and the large amount of possible chemical modifications potentially allow designing high voltage/capacity OMB electrode materials, the large search space requires efficient exploration of potential molecular-based electrode materials by rational design strategies on an atomistic scale. By means of density functional theory (DFT) calculations, we provide insights into possible strategies to increase the voltage by changes in electronic states via functionalization, by strain, and by coordination environment of Mg cations. A systematic analysis of these effects is performed on explanatory systems derived from selected prototypical building blocks: five- and six-membered rings with redox-active groups. We demonstrate that voltage increase by direct bandstructure modulation is limited, that strain on the molecular scale can in principle be used to modulate the voltage curve and that the coordination/chemical environment can play an important role to increase the voltage in OMB. We propose molecular structures that could provide voltages for Mg insertion in excess of 3 V.

## Introduction

The development of smart grids, renewable energy sources (Dunn et al., 2011) and electro-mobility (Lu et al., 2013) are just a few fields that demand improved electric energy storage technologies. New solutions, which meet the growing demands of scalability, better performance, and lower costs, should also be sustainable and non-toxic. Secondary metal ion battery technologies are at the forefront of current electrical energy storage research as they can provide high energy density, be used in mobile as well as stationary/large scale applications and could last thousands of charge-discharge cycles (Hameer and van Niekerk, 2015). Metal ion batteries are increasingly used not only in mobile devices but also to palliate load-level balancing of solar- and wind power plants. To fully access the benefits of this type of technology, many aspects need to be improved such as slow ion diffusion kinetics limiting the rate performance or capacity and cyclability limitations as well as user and environmental safety (Goodenough, 2012).

Many open challenges are of course materials related; specifically, the active electrode material's interaction with metal-ions, e.g., lithium (Li) (Diouf and Pode, 2015), counter ions (Chen et al., 2018) or common electrolytes critically dictates the materials performance. Today, various types of active electrode materials (from here on referred to as electrode for brevity but to be distinguished from contact electrodes) are known that can be manufactured from various sources. Examples are layered structures (Kulish et al., 2014; Sun et al., 2016; Ortiz-Vitoriano et al., 2017), metal oxides (Liu et al., 2017), polymers (Nishide et al., 2004; Koshika et al., 2010; Muench et al., 2016), or metal-organic frameworks (Xue et al., 2016). Electrode materials can differ in their redox-active centers—like compounds with oxygen or sulfur atoms, or functional groups. Thus, the search for high-performance electrode materials extends over a huge search space of materials, and much effort was made and will continue to be necessary to explore it further to find new materials leading to improved battery performance (Mohtadi and Mizuno, 2014; Yabuuchi et al., 2014; Kim et al., 2015a; Wang et al., 2015; Muench et al., 2016; Mauger et al., 2017; Leisegang et al., 2019).

Organic electrode materials have gained much interest (Liang et al., 2012; Lee et al., 2018; Manzhos, 2019) as their production can be sustainable (Xu et al., 2008; Zeng et al., 2014), and non-expensive; they can be recyclable, non-toxic and have a low carbon footprint at the same time (Liang et al., 2012; Deng et al., 2013). They can rely on well-developed chemistries and industrial base. Indigo carmine is an example of a well-known organic material that can be used as an electrode (Yao et al., 2010). Moreover, organic materials are most suitable for sustainable energy storage technologies and do not defeat the purpose of renewable energy technologies (i.e., sustainability and scalability). For instance, load leveling of electric grids in which a fluctuating amount of excess energy from renewable energy sources must be stored and released. In addition, it was shown that by some measures (e.g., extremely high cycling rate) organic materials can outperform commercially available inorganic electrode materials (Koshika et al., 2009). Development of organic electrode materials for metal ion batteries has picked up momentum in recent years, both through experimental and theoretical studies (Stephan and Nahm, 2006; Bhatt and O'Dwyer, 2015; Chen and Manzhos, 2016a; Padhy et al., 2018; Tripathi et al., 2019) Moreover, many of their properties including redox activity can be tuned (Oltean et al., 2016; Chen et al., 2017) by molecular design allowing an excellent adaptability for particular technological fields. Remaining challenges to achieve better performance in e.g., capacity, voltage, power, rate capability, and cyclic stability might be solvable.

Among all secondary metal ion battery types, only Li ion batteries are widely used, for instance in commercially available lithium cobalt oxide based batteries due to their high energy density. However, lithium as a resource is very unevenly distributed and is relatively scarce, whereas many other metallic elements, which can be used for so-called post-lithium batteries, like sodium (Na), magnesium (Mg), or aluminum (Al) are abundant (Leisegang et al., 2019). Much research effort has therefore been directed to make use of alternative metal ions that could replace Li. Na ion is a suitable candidate for these batteries because Na is abundant, cheap and it has a similar valance shell structure as Li, i.e., one unpaired valence *s* electron (Ratnakumar et al., 1990; Wenzel et al., 2011; Lu et al., 2012; Slater et al., 2013). Often, sluggish kinetics, low voltage and low capacity limit the success of Na ion batteries. Moreover, the same inorganic host materials used in Li ion batteries may be unsuitable for reversible Na insertion. Well-known examples are graphite and silicon wherein lithiation but not sodiation can be achieved (Stevens and Dahn, 2001; Ge et al., 2012; Malyi et al., 2013). Nonetheless, organic and inorganic materials for Na ion electrodes were discovered that can outperform commercially available Li ion batteries in some key performance parameters (Zhao et al., 2016; Lee et al., 2018; Padhy et al., 2018).

Multivalent charge carriers like Mg^2+^ or Al^3+^ (Mohtadi and Mizuno, 2014; Leisegang et al., 2019) could theoretically double and triple the capacity per carrier of the active material compared to Li^+^ and Na^+^ as two (for Mg) or three (for Al) electrons would be transferred per active cation instead of the one electron from Li or Na. In particular, metallic Mg as anode material is interesting in this regard due to further benefits that are (i) being environmentally friendly, (ii) non-toxic, (iii) biodegradable and (iv) allowing for dendrite-free deposition (Gregory, 1990). For inorganic materials, examples for electrode materials for the storage of multivalent active cations are vanadium oxides or manganese oxide and Prussian blue analogs that work with several metal ions including Mg^2+^ (and others; Hayashi et al., 2004; Xu et al., 2015; Tojo et al., 2016; Kulish et al., 2017a,b; Koch and Manzhos, 2019). However, the larger charge on the ions leads to significant polarization and has negative effects on the kinetics/diffusion rate in inorganic solids (Kulish and Manzhos, 2017). Organic electrode materials could in principle achieve fast kinetics with multivalent cations compared to inorganic materials due to larger atomic spacing (Canepa et al., 2017) but it is still a challenging field of research. Few multivalent organic electrode materials with sufficiently high voltage for a cathode were reported, that are, e.g., quinones such as anthraquinones providing voltages of ca. 2 V as Mg ion battery cathodes (Sano et al., 2012; Zhao-Karger et al., 2013; Bitenc et al., 2015, 2016; Pan et al., 2016a,b). The low voltage is a key problem in multivalent batteries and is a direct consequence of multivalence: the average voltage *V* to achieve a given state of charge is

(1) $$\begin{array}{r} V=-\Delta G/ne, \end{array}$$

where Δ*G* is the respective free energy change, *e* the electron charge and *n* the number of charges transferred per elementary reaction (i.e., *n* = 2 with Mg; Urban et al., 2016). That is to say, for a cathode, a very high Δ*G* (i.e., high Mg-host interaction energy) should be achieved while preserving stability of the electrode material and reversibility of Mg insertion. Many organic materials that are redox-active with Na or Li atoms such as the well-studied carboxylates (Park et al., 2012; Renault et al., 2013; Sk and Manzhos, 2016) do not result in strong enough interaction that would allow using them in Mg ion batteries.

A systematic approach is required to understand and improve organic electrodes for multivalent Mg (ion) batteries. Rational design based on atomic insights from *ab initio* computations is critically important for this purpose and in particular to understand and systematically explore the connection between material properties, such as electronic structure, interlayer spacing and crystallinity, and the redox processes in materials (Miroshnikov et al., 2016; Seo et al., 2016; Araujo et al., 2017; Molaei et al., 2017). Different design approaches should be tested computationally for molecules and for solids to modulate the interaction strength with inserted metal ions and the voltage (Kulish et al., 2017a). For instance, *p*-doping, i.e., the creation of a “hole” state in the electronic structure in the vicinity of the valence band maximum (and below the lowest electronic conduction state) increases the binding strength of Li in aluminum doped silicon and of Li on boron-doped disodium terephthalate by ca. 1 and 2 eV, respectively (Legrain and Manzhos, 2015; Lüder et al., 2017a,b). Adding/replacing electron-withdrawing groups, e.g., F atoms or cyano (CN) groups, in a molecular structure can also increase the voltage by increasing the binding strength to the active cation (Chen and Manzhos, 2016c; Chen et al., 2018). Doping is only one way to affect the performance of batteries and it is particular in that it directly addresses the electronic structure of a material. Other modifications can aim to alter strain effects, the chemical surrounding, or a combination of them. In this work, we focus on materials that work by reduction, i.e., in materials in which the lowest unoccupied molecular orbital (LUMO) of the electrode determines the redox potential. For materials that work by oxidation, the HOMO (highest occupied molecular orbital) determines the voltage and effective strategies there can be different (Geniès et al., 1989; Chen and Manzhos, 2016c; Rodriguez-Pérez et al., 2016; Chen et al., 2018).

In this study, we explore conceptual solutions for high voltage organic molecule based Mg (ion) electrode materials operating by reduction using Density Functional Theory (DFT) calculations. We organize the paper as follows: firstly, we explore conceptually the limitations of the approach of increasing the voltage in organic Mg-ion batteries working by reduction only by direct electronic effects. We show that this mechanism has substantial limitations on achievable voltages and possibly leads to unwanted side reactions. With the help of small organic molecules consisting of five- and six-membered rings with redox-active carbonyl groups that we employ as model systems, we show the extent to which expected voltage can be modulated by design of electronic structure such as the use of substitutional doping (Lüder et al., 2017a,b). We study how much the electronic effects can be separated from others for these systems. Secondly, the influence of strain (linear, rotational (bending) and dihedral strain) on the expected voltage with a molecule is elucidated (Chen and Manzhos, 2016b). Because of the limitations of design by the modulation of the electronic states, strain design and the effective use of it to increase the interaction strength to metal ions can potentially further contribute to increase in voltage. Third, we discuss a new design aspect that addresses the arrangement of the coordination centers such as ring linking and fusion to design cation environments with high redox potentials.

## Methods

The Gaussian (Frisch et al., 2016) package was used to perform DFT (Hohenberg and Kohn, 1964; Kohn and Sham, 1965) calculations. The B3LYP functional (Becke, 1993) and the 6–31+G(d,p) basis set were used on all atoms. This basis set provides sufficiently accurate energies for the attachment of alkali atoms on organic molecules due to the inclusion of diffuse and polarized functions.

The formation energy per metal atom of *m* (*m* = 1, 2, 3, …) metal atoms (e.g., Mg) and one molecular complex was computed from the total energy of *m* metal atoms attached to the complex at their fully relaxed geometry ($E_{r}^{mol+M}$) and the total energies of *m* isolated metal atoms (*E*^*M*^) and the isolated and relaxed molecule ($E_{r}^{mol}$).

(2) $$\begin{array}{r} E_{ad}=\left(E_{r}^{mol+M}-E_{r}^{mol}-m\;E_{\;}^{M}\right)/\;m \end{array}$$

From the formation energy, the voltage *V* vs. Mg^2+^/Mg can be estimated according to Ceder et al. (1997) and Urban et al. (2016)

(3) $$\begin{array}{r} V=-\frac{\left(E_{ad}-E_{coh}^{M}\right)}{en} \end{array}$$

which relates the formation energy of the Mg-molecule complex to the cohesive energy of the metal $E_{coh}^{M}$ in its bulk phase. For Mg $E_{coh}^{M}$ is taken from the literature as 1.51 eV (Kaxiras, 2003; Kittel, 2005), and the charge (*en*) transferred in the redox process with *n* being the number of electron charges per cation is taken as two *e*^−^ for Mg. This method of estimating the voltage approximates the Gibb's free energy (Equation 1) by neglecting entropy contributions as well as effects from vibrations. Nonetheless, the approach based on electronic energies computed by DFT to compute voltages has been successfully used before (Urban et al., 2016). It can also be used for molecular systems with accurate reproduction of experimental voltages with models of molecular solids, while in isolated molecular models the neglect of environmental effects causes an underestimation of the absolute values of the voltage on the order of 1 V but allows reliable comparative calculations between different molecular structures (Sk and Manzhos, 2016; Chen et al., 2017; Manzhos, 2019). This is also the approach used here—we use molecular calculations to compare the effect of molecular building blocks on the strength of interaction with Mg atoms and on voltage vs. Mg^2+^/Mg.

The binding strength of a Mg atom to the electrode material should exceed the cohesive energy of metallic Mg, or else the so-called plating will occur. When the binding energy is equal to the cohesive energy, the equivalent voltage based on Equation (3) would be 0 V. When there is no binding between the molecule and the Mg atom the theoretical voltage based on Equation (3) is formally −0.755 V and is negative as long as the binding strength is weaker than $E_{coh}^{M}$ of metal *M*. Negative voltage would of course not be observed experimentally but will be used in what follows for formal comparison between molecules. Also, as single-molecule models underestimate the voltage, molecular systems with formally negative voltages might be of practical interest.

The linear (achieved through elongation of bond lengths, e.g., between Mg and the redox-active parts or intramolecular bonds), rotational (caused by bending) and dihedral (i.e., through changes in dihedral angles) strain energy *E*_*s*_ of a molecule, that is caused by the attachment of the Mg atoms or structural deformations induced otherwise, is computed as the energy difference between the molecular structure it assumes when the metal atom is attached (but single point calculations are done without the metal atom) or the deformation is applied ($E_{f}^{mol}$) and the isolated fully relaxed molecule or the molecule before the deformation ($E_{r}^{mol}$)

(4) $$\begin{array}{r} E_{s}=E_{f}^{mol}-E_{r}^{mol} \end{array}$$

Linear strain is created by pulling a molecular complex apart along a chosen direction. The relaxation of the structure is performed on all but a minimal set of frozen atoms to control the strain direction. Bending strain generated by pushing a set of atoms within a molecular complex closer to each other and keeping their coordinates fixed during structural relaxation while all other atoms fully relax. Dihedral strain is set by freezing the coordinates of atoms fixed during structural relaxation that define a dihedral angle between connected rings. In this study, all deformations that lead to strain other than by Mg attachment are achieved by keeping atomic coordinates fixed during structural relaxations.

## Results and Discussion

### Limits of Electronic Effects for Voltage Increase in Organic Mg (Ion) Electrode Materials

It was shown in several studies that the energies of the LUMO significantly correlate to the voltage achieved with materials operating by reduction as the electron from the attached alkali atom, e.g., Li, will occupy the LUMO of the molecule (Burkhardt et al., 2013; Liang et al., 2013; Kim et al., 2015b, 2016; Lüder et al., 2017a). Specifically, a stabilization of the LUMO energy of comparable systems, for instance by substitutional doping, show strong correlations to voltage increase. When one uses in this model the LUMO energy before molecular reduction, we address this model as static. However, the precise value of the voltage increase will depend on several factors due to structural and electronic relaxations during the redox reaction. For Li-ion electrode materials, the energy difference between the energy (eigenvalue) ε_*Li*2*s*_ of the 2*s* level in Li (or the Fermi level in the metallic anode if it is used as a reference) and the LUMO of the molecule will lead to a gain in total energy (and free energy) that will drive the reaction and largely determine the voltage, see Equation (1). According to this model, any change in the LUMO energy will affect the voltage, i.e., a lower LUMO will lead to a larger voltage. In our previous studies (Lüder et al., 2017a,b; Chen et al., 2019), we showed that substitutional *p*-doping can be used to lower the LUMO of a molecule. The LUMO shifts to a lower energy and therefore the energy gained in the redox reaction is increased, which was observed for different prototypical organic Li- and Na-ion electrode materials.

**Figure 1:**
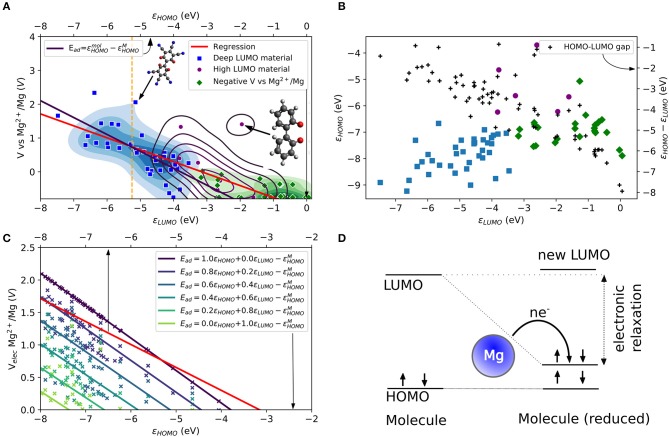
**(A)** Computed LUMO eigenvalues and theoretical voltages vs. Mg^2+^/Mg for a set of small organic molecules. The molecules are categorized into three groups (blue, purple and green areas and data points) by a k-means algorithm. A linear regression (red curve) is compared to an electronic approximation (black curve) to Equation (2). The top axis refers to a comparison based on the electronic effects assuming a LUMO relaxation reaching HOMO values. **(B)** Comparison of the relation between HOMO and LUMO eigenvalues, and HOMO-LUMO gaps for the three categories in the dataset. **(C)** Comparison of the linear regression (red curve) from (a) different LUMO relaxation values in the electronic approximation to Equation (2). **(D)** Schematic of the LUMO relaxation process during the redox reaction. For a Mg atom, two electrons are transferred (i.e., *n* = 2). Arrows indicate electrons with spin-up and spin-down. Color code is H (white), C (gray), N, (blue), O (red), F (light blue), Mg (yellow), and S (dark yellow) here and below.

The same mechanism can be explored for Mg atom in which the role of the HOMO energy level of the Mg atom (ε_*Mg*3*s*_) and the electronic structure of the tested molecules are evaluated. In Figure 1A, the theoretical voltage computed for the attachment of one Mg atom is plotted vs. LUMO energy for a large number of tested molecular structures. The structures are shown in the Supplementary Material in Figures S1–S5. One observes that there is indeed a correlation, albeit somewhat loose, between the voltage and the LUMO. We highlighted different areas in Figure 1A. The areas are obtained by a *k*-means cluster finding algorithm on the data. The separation in three groups is based on the assumption that the tested molecules can be separated into non-working, low voltage and high voltage electrode materials. The algorithm employed the HOMO and LUMO energies, as shown in Figure 1B together with the HOMO-LUMO gap (black points) as features. The three areas can be roughly distinguished as molecules with higher voltages that do correlate well to very low LUMO energies (blue area, “deep LUMO”), molecules with high LUMO energies and formally negative voltage (i.e., unsuitable as active electrode materials, green area), and molecules with moderately high LUMO energies (magenta). In Figure 1A, the scatter of voltage-LUMO data is not surprising as there are other factors than bandstructure energy (we will liberally use “bandstructure” as set language even when talking about molecules).

The red curve in Figure 1A shows the linear regression of the LUMO-voltage data. The curve shows the well-known correlation between decreasing the LUMO energy and increasing the voltage, which is frequently observed for Li- and Na-ion electrode materials (organic and inorganic). Here we see that such correlation also largely holds for Mg attachment as well. It should be noted that the LUMO energies refer to the values of the single molecule without metal atom attached. The curve shows that a LUMO energy of <-3 eV is needed for a working anode as implied in Equation (3). Furthermore, the curve also reveals that for high voltage electrodes (more than 2 V) a LUMO energy of <-8 eV would be required from this mechanism.

The simplified picture above is based on the frozen state approximation. Relaxation effects could be significant. To account for the energy level relaxation, the energy level of the molecular LUMO, which becomes occupied by the valence electrons of the metal atom, may shift to lower energies. This process was observed, for instance, for the boron-doped coronene molecule under Li and Na attachment (Lüder et al., 2017b). Here, we include the LUMO relaxation effect after reduction and assume that the shifted LUMO will not decrease to energies below the HOMO energy. Thus, the change of free energy based on the electron transfer from metal to an organic electrode (which is the net effect, even though the electrons transfer through the external circuit) can increase by the electronic relaxation of the LUMO energy during the redox reaction. Of course, the most significant change is achieved when the LUMO energy relaxes to values close to the HOMO energy. This effect becomes less significant for the redox process with decreasing LUMO energy level as displayed in Figure 1B due to the decrease of the HOMO-LUMO gap (black dots, right axis) or reduced relaxation. The figure also shows the three different types of materials determined by the *k*-means classification, which are distinguishable by their HOMO and LUMO.

Figure 1C compares the regression of the computed data with the extended electronic model (i.e., including LUMO energy relaxations) illustrated in Figure 1D at different amounts of state relaxations. The free energy that enters the computation of the voltage [or the *E*_*ad*_ of Equation (3)] is replaced by a weighted energy difference between HOMO and LUMO energy of the molecule and the HOMO energy of the Mg atom (−5.3 eV). It reads

(5) $$\begin{array}{r} E_{ad}={a\;\varepsilon }_{HOMO}^{mol}+b\;\varepsilon_{LUMO}^{mol}-\varepsilon_{HOMO}^{Mg} \end{array}$$

in which the weight factors satisfy *a* + *b* = 1. The sets of data points (indicated by different coloring) correspond to different weighting factors of the computed data shown in Figures 1A,B and the solid lines to their resulting linear regressions. The values of HOMO and LUMO energies are regarded in this model as a key determining factor of *E*_*ad*_ and therefore give the limits of the electronic model. This model results in two extreme cases for the computed voltage that can be compared to the computed data. However, it must be noted that the calculated data (Equation 3) use the total energy changes obtained by DFT, while the estimates of the model are based on eigenvalue changes. Their sole purpose is to elucidate possibilities and limitations of voltage modulation by electronic effects. It is a measure of the gain in energy solely due to bandstructure (equivalent to a charge transfer in frozen orbitals), while the computed voltage data include further relaxation channels (e.g., charge distribution, transfer, bond length changes) that affect the change in free energy, too. The energy of the LUMO defines one electronic limit (light green curve). The corresponding curve in Figure 1C is far below the linear regression of the computed data (red curve). For this case, working voltages (*V* > 0) require HOMO energies of <-7 eV because the redox process will not benefit from any LUMO relaxation in the static case. With increasing electronic relaxation, i.e., the LUMO after reduction approaching values closer the HOMO energy, the curve approaches voltages close to 2 V. The other limit (black curve) is yielded by the HOMO energy, i.e., electronic relaxation where the occupation of LUMO makes its energy approach the HOMO. The resulting curve intersects with the regression curve of the computed data at ca. −5.5 eV while it overestimates voltages below and underestimates voltages above this energy. The same curve is overlaid in Figure 1A as black curve and it can be regarded as the electronic limit. From the figure, it becomes clear that the LUMO must be very deep to result in a positive voltage. In this model, the LUMO must be lower than ca. −8 eV to achieve voltages larger than 2 V.

Besides the apparent 2 V limit by electronic structure design, two more observations should be mentioned: First, neither the extended electronic model nor the static model offers a solution to the intrinsic limitation of electronic structure design for Mg electrode materials. Instead, they can be understood as different mechanisms (or point of views) in the redox process governed by electronic effects. While the static model requires a low LUMO for high voltages, this can also be achieved with a high LUMO material but with sufficient relaxation effects (requiring a low energy of HOMO). Second, while the electronic limit based on the HOMO energy appears similar to the regression curve based on the computed data, the actual LUMO relaxation does not reach energies close to the HOMO of the molecule before reduction. Thus, the electronic model is compensating for other effects that appear simultaneously, as mentioned above.

As a reference of realistic and technologically relevant materials operating by reduction, we consider the LUMO energies of tetracyanoethylene (TCNE) which is known for its electron-accepting property, i.e., a very low LUMO energy in a stable molecule associated with a large absolute electron affinity (Pearson, 1986). The LUMO energy of TCNE is shown as vertical dashed orange line at −5.25 eV (Chen and Manzhos, 2016b) in Figure 1A. A lower LUMO would in principle results in stronger reduction activity. Besides the difficulties of designed materials with extremely low LUMO energies, such materials would be extremely unstable. Even as packing effects in a solid state are expected to result in a somewhat higher voltage than computed from a single-molecule model (Manzhos, 2019), this clearly shows the severe limitation of the strategy of LUMO design in the case of Mg ion batteries even as it served well organic material design for Li and Na ion batteries.

Electrodes in organic Mg and Mg-ion batteries often use carbonyl groups, which create the danger of parasitic MgO formation. The formation energy of MgO of 3.07 eV implies an equivalent of about 0.75 V vs. a metallic Mg anode. This estimate gives a target for the minimal voltage that a molecule should achieve to avoid loss of capacity because of redox reactions resulting in voltages above these values, the formation of MgO could be avoided.

Figure 1 contains data for several classes of small organic molecules with a ring structure, including benzene, 1,4-benzoquinone, biphenyl and biphenylene. They include the unmodified molecules, and, e.g., carbonyl, CN- and F-functionalized derivatives; details can be seen in SI. These functional substituents are known as electron-withdrawing groups. Thus, any substitution in benzene, benzene-based molecules or similar ring structures is expected to result in an electro-positive part (e.g., C atoms) and a more electro-negative part (i.e., the functional groups). Besides the redistribution of electronic charge, stabilizing effect on HOMO and / or LUMO energy and therefore changes in the interaction strength between a metal cation and the redox-active molecule can be anticipated, potentially with an increase in voltage. This was observed before, as mentioned earlier, in organic Li- and Na-ion electrode materials. However, the CN/F-functionalized benzene results cannot confirm this for organic Mg (and in principle Mg-ion) electrode as it did not significantly increase the interaction with Mg. In addition, we also investigated other halogen functionalization of benzene (e.g., Cl and Br). Our results indicate that electronic effects achieved by functionalization of materials can lead to small modulations of the voltage if a sufficient number of redox-active groups such as carbonyls are present.

On the other hand, a careful inspection of the data shown in Figure 1A reveals a few exceptional prototypical molecules (indicated by arrows and given molecular structures of biphenyl derived molecules). These molecules have (i) a LUMO energy higher than the one of TCNE and are therefore expected to be stable and (ii) their computed voltages are far above the regression curve and even beyond the electronic limit indicating that other processes in these molecules occur that strongly benefit the redox mechanism. Identifying the mechanism behind a higher computed voltage in these molecules and deriving molecule design strategies based on them will be discussed in the following.

### Effect of Structural Modifications Leading to Strain

Reversible structural modifications can influence the total energy of a molecule as well as electronic states. HOMO, LUMO energies and the HOMO-LUMO gap can be modulated by strain. Both, changes in the total energy and that of individual electronic states can in principle lead to changes in the interaction strength between molecules and Mg atom(s). Thus, it will affect the voltage according to Equations (1–3). However, the direct application of this idea for molecular-based electrode design and practical applications in batteries is not frequently explored.

To efficiently modify voltage curves, the changes of the total energy must mainly affect the mentioned interaction between the metal atom and the molecule while the intra-molecular changes and their associated energies are small, or vice versa. When the structural modifications induce total energy changes of similar size and same direction (that means endo- or exothermic), the resulting energy difference in the redox mechanism will cancel out according to Equation (1).

We distinguish between three principal structural changes: linear stretch, bending of the molecular plane, and dihedral rotation. For these three strain designs, we discuss prototypical systems.

#### Linear Strain

Here we study a system of a Mg atom coordinated to two molecules by two carbonyl groups, to account for the transfer of two electrons (one to each group). In a system of linear geometry in which two molecules with a carbonyl group surround a Mg atom, one way to introduce linear strain is by pulling either one of the molecules or both molecules simultaneously away from the Mg atom. Linear strain can also be introduced by elongating the bonds within a molecule. However, our investigation showed that for the latter case, effects on the LUMO or HOMO energy position are minor, and the binding strength to Mg atoms is barely affected. Moreover, the strong covalent bonds within organic molecule prevent a significant elongation by forces with technologically relevant magnitude. Thus, we focus on the approach of changing intermolecular geometry, i.e., the Mg-O bonds.

**Figure 2:**
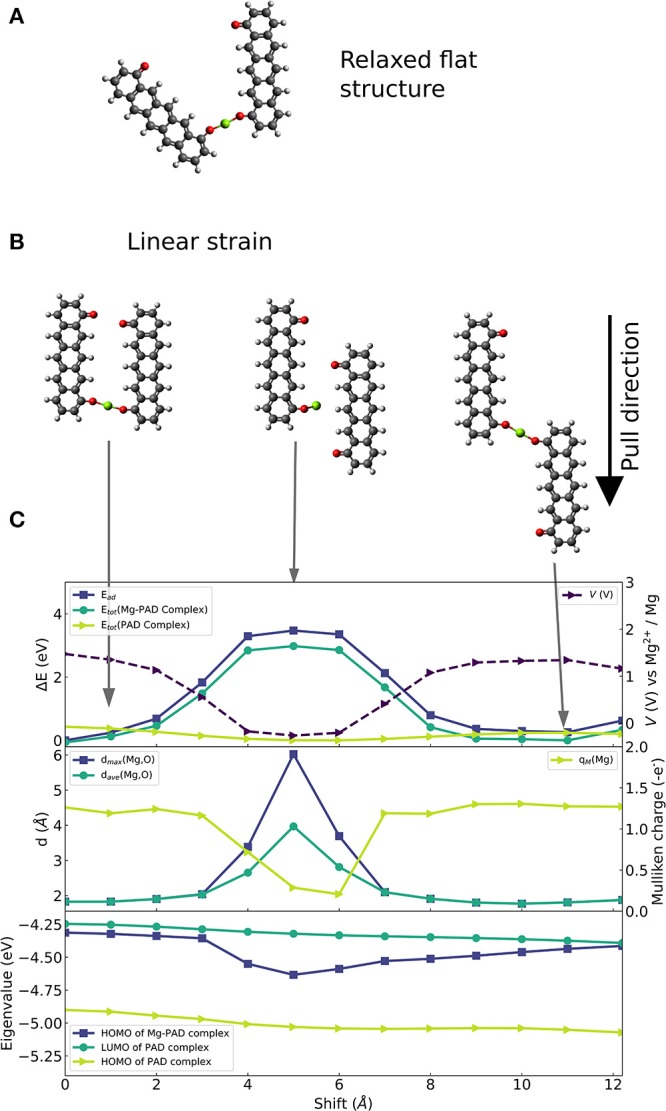
Constrainted calculation on 6-membered ring-fused PAD structure with two carbonyl groups per molecule. **(A)** Optimized geometry of the planar system. **(B)** Selection of strained configurations in which one of the PAD molecules is parallelly shifted with respect to the other molecule: (left) Initial structure with parallel alignment of the PAD molecules shown in **(A)**, (middle) configuration with the highest strain energy equivalent, (right) configuration with final shift with a similar O-Mg-O bond as in initial structure. **(C)** Overview of electronic changes and properties during the shift: (top) Change of reaction energy (left axis) of Mg insertion (dark blue, square), change of energy of the complexes with (green, circle) and without Mg atom (light green, triangle), as well as computed voltage during parallel shift (right axis, dashed curve). (Middle) Maximal (blue, squares) and average distance (green, circles) between nearest two O atoms and the Mg atom; Mulliken charge on the Mg atom (light green curve) is given on the right axes. (Bottom) Eigenvalues of HOMO before and after Mg attachment (light green and blue, respectively) and eigenvalues of LUMO before Mg attachment (green).

The chosen molecular complex that is used to investigate the effects of linear strain on voltage modulation is shown in Figure 2. We select a ring-fused linear molecule consisting of five six-membered rings. The molecule was laterally functionalized with two carbonyl groups with a distance between them of ca 9.8 Å, i.e., forming a pentacenedione (PAD) isomer. To systematically investigate the effect of linear strain on voltage modulation at the molecular scale and to provide technologically relevant insights, constraint geometry optimizations are required imposing constraints on and offering control of degrees of freedom. We restrict our analysis to a planar configuration of two PADs. This allows us to directly probe the correlation between atomic geometry, electronic effects, and the applied strain. Figure 2A shows the equilibrium structure of this (planar) configuration of the complex formed by a Mg atom and two 4,8-pentacendione molecules. We formed a series of geometries (of which three selected cases are shown in Figure 2B) in which the intramolecular degrees of freedom are relaxed, and the molecules are made slide in parallel to and past each other in the same plane. The initial structure in Figure 2B has the same Mg-O bond lengths as in the equilibrium structure. The middle figure of this panel shows the strained structure at an intermediate strain stage. The right panel of Figure 2B shows the most strained structure with a displacement of ca 12 Å and an O-Mg-O bond which is again similar to the one of the initial structures.

Figure 2C shows (top panel) changes in the binding strength of the configuration with the strongest binding, and changes in the total energy of the structure with and without the Mg atom (middle panel); binding distances to and Mulliken charges of the Mg atom (bottom panel); HOMO and LUMO eigenvalues of the Mg-complex and the complex without Mg atom. At the geometry with the largest strain energy shown in the middle of Figure 2B, there is a significant decrease in binding strength, up to 3.5 eV. The decrease in binding strength translates into a voltage reduction of 1.75 V. At this stage, one Mg-O distance increases to 6 Å, which is too long for any covalent bond, see middle panel of Figure 2C. Hence, the complex has lost one of the two bonds while the other Mg-O distance remains at a typical value for strong bonds of ca. 1.9 Å. In addition, the electron charge transfer from Mg to the organic part of the molecule is reduced, which is seen in a drop of Mulliken charge on Mg to almost 0. Then, the decrease of binding strength can be explained by the loss of a Mg-O bond.

In contrast, the average and maximal distance of Mg-O are <2 Å (that is for average and max.) for maximal binding strength, and the Mulliken charge on Mg is ca. +1.3 |*e*| which corresponds to transfer of two electrons. Besides, the electronic structure (HOMO and LUMO) of the complex without the Mg atom (green curves in the lower panel of Figure 2C) is only slightly affected by strain. The LUMO relaxation effect is given by the energy difference between the LUMO energy of the molecular complex before Mg insertion (dark turquoise curve) and HOMO energy of the Mg-molecule complex (blue curve). It is very interesting that this relaxation effect shows a significant increase that correlates with the strain dependent weakening of the binding strength. This suggests that both effects, one being a charge transfer and bond release driven—the other being an electronic effect, act in the presented case in opposite directions.

At the final stage of the applied strain, the average and maximal Mg-O distance, Mulliken charge and binding strength reach values similar to the ones given at the initial stage indicating possible reversibility. Moreover, they imply that a key factor to efficiently design molecular systems, that can employ strain-based voltage modulation, is the ability to control the distance between Mg and the redox-active groups, and the number of formed Mg-O bonds. This suggests that the design of coordination of Mg to several redox-active groups is important; we will address this issue in the section Design of Coordination Environment of Mg Cations. However, the seen strain effects mainly reduce the voltage. Thus, the charging voltage of an organic electrode material can be reduced by applying linear deformation assuming the microstructure of the material supports this reversibly.

The results of the linear strain are in agreement with basic chemistry: at large distances no bonds are formed while at shorter distances the hybridization of states causes the formation of bonds (and consequent charge transfer leading to more ionic interactions). It should be noted that there is no strong correlation between the changes of the LUMO of the molecule only complex, and that changes in *V* are almost binary to situations when Mg binds to one and to two redox centers—preferring two redox centers. For situations in which several redox centers surround the Mg ion—such as in a solid, a large molecule or several molecules—controlling the distances and amount of redox active centers binding to Mg may provide a way to increase voltages.

#### Bending Strain

We analyze the effect of bending in molecular complexes in which Mg binds to two neighboring molecules. Similarly to the linear strain section, constrained geometry optimizations were performed to investigate the effects of a bent molecular structure on the binding strength between organic electrode materials and the inserted Mg atom.

**Figure 3:**
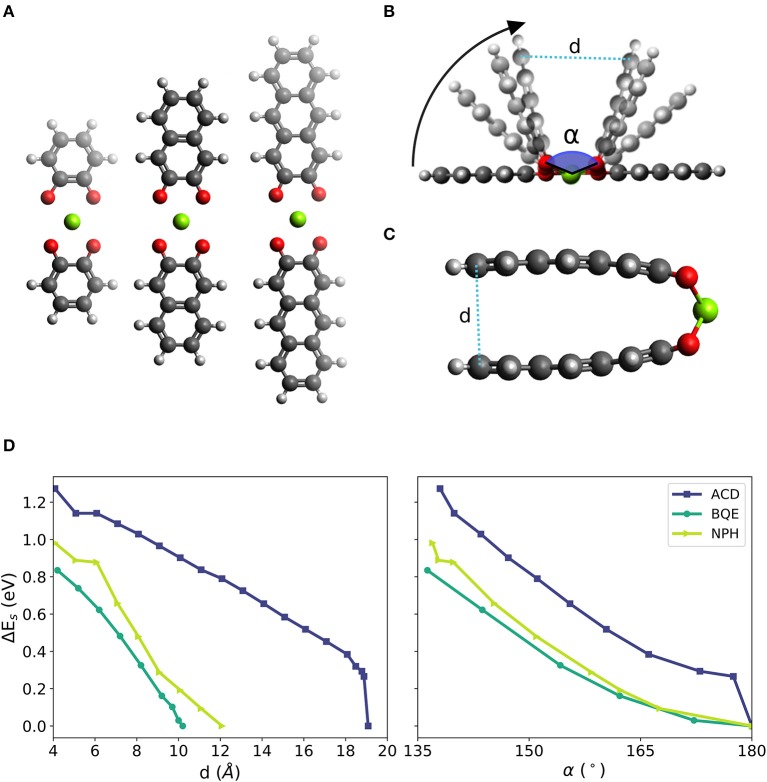
**(A)** Linear configurations of two prototypical molecules of different size (from left to right: one, two and three fused benzene-like rings, i.e., complexes based 1,2-benzoquinone (BQE), 2,3-naphthalenedione (NPH) and 2,3-anthracenedione (ACD), respectively) with two neighbored carbonyl groups of each molecule surrounding one Mg atom. **(B)** Schematic of the bending simulations with bending angle α defined by the O-Mg-O bonds across the two organic molecules in the structure and distance d between the outer parts of the complex. **(C)** Example of final bending configuration of the NPH complex with almost parallel molecular planes of the two ACD molecules in the complex. **(D)** Change of strain energy during bending as function of distance d (left) and as function of the bending angle α (right).

We chose the 1,2-benzoquinone (BQE), 2,3-naphthalenedione (NPH) and 2,3-anthracenedione (ACD), which are molecules similar to PAD but with a reduced number of fused six-membered rings. BQE, NPH and ACD have one, two and three fused 6-membered rings, respectively. In each initial structure of the molecular complexes, two identical molecules from a linear complex with a Mg atom at its center. A planar configuration was imposed by symmetry during relaxation to simplify the evaluation of the bending effect (the NPH and ACD complexes have dihedral angles different from 0° at their lowest energy configurations). The complexes in their fully relaxed planar form are shown in Figure 3A. The bending was initialized by decreasing the distance *d* between the outer most carbon atoms (called the edge of the molecule) in steps of 1 Å until a distance of ca. 4 Å was reached. Four different bending stages of Mg-di-NPH are shown Figure 3B in which curved arrows indicate the bending direction. An example of a final bending configuration is shown in Figure 3C for the ACD Mg complex with almost parallel molecular parts.

Figure 3D shows the increase in strain energy (*E*_*s*_) when the Mg complexes are forced into a bent structure. All other atoms but those at the edges are allowed to fully relax including the bonds between the Mg atom and the carbonyl groups. Thus, any effect leading to a weakening of this bond (i.e., an increase of *E*_*s*_ or a decrease of *E*_*ad*_) is expected to be accurately represented. After a steep increase at the beginning, the bending curves for the three studied complexes yield similar values of 0.8 to 1.2 eV (corresponding to a voltage decrease of ca. 0.5 V vs. Mg^2+^/Mg) when the two molecules reach almost parallel alignments (at a distance of ca. 4 Å), shown in Figure 3D, left panel. This indicates that the bending strain is not (strongly) depending on the size of the molecule but depends on the bond angle formed between O-Mg-O, shown in Figure 3D, right panel.

#### Dihedral Strain

The effect of changing a dihedral angle in a molecular structure on the interaction strength between Mg and an organic molecule can be tested similarly to the effect of bending. However, single fused-ring molecules like biphenylene or naphthalene exhibit strong restoring forces when the molecular structures are twisted. Neither electronic nor structural changes were found to be large enough under this deformation to result in significant voltage changes for these molecules. Thus, we focus on molecular prototypes like biphenyl-derived systems (as seen in the section Limits of Electronic Effects for Voltage Increase in Organic Mg (Ion) Electrode Materials) with a bond between ring structures that can act as a rotational axis. Although biphenyl is known for its sterically hindered full dihedral rotation of the two phenyl rings, we expect to see a rather low energy increase for a wide range of dihedral angles. The mentioned bond on which the dihedral rotation is performed is referred to as the rotation axis and the change of dihedral angle as Δγ.

**Figure 4:**
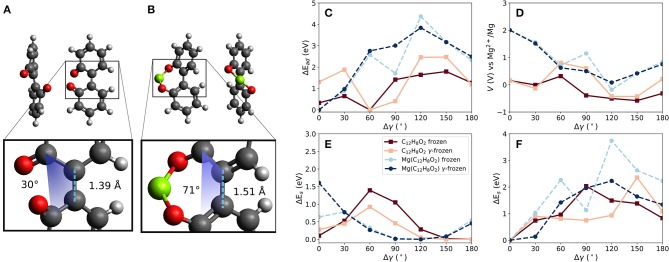
Effects of dihedral angle change in a biphenyl-like molecule with two carbonyl groups (BipO2) at connected phenyl-like rings. Differences in bond lengths between phenyl-like rings and dihedral angle γ in **(A)** BipO2 after Mg detachment and **(B)** MgBipO2, i.e., BipO2 with one attached Mg atom. **(C)** Change of interaction strength between Mg and the molecule for frozen and partly frozen, i.e., γ-frozen, relaxations based on the optimized BipO2 structure, as shown in **(A)**, red curves, and based on MgBipO2, as shown in **(B)**, blue curves. The computed voltages are shown in **(D)**. The strain energies given in **(E)** and **(F)** are computed without and with one attached Mg atom, respectively.

Two carbonyl groups were introduced into the biphenyl molecule (C_12_H_8_O_2_, referred to in the following as BipO2) shown in Figure 4A. The carbonyl groups are on opposite phenyl rings. For this molecule, two principal configurations of the molecule are possible—one with the two carbonyl groups close to each other (as shown in the figure) and the other with them far from each other. This is obtainable by a dihedral rotation. The larger separation of the carbonyl groups is energetically preferred by 0.3 eV. Bond lengths between atoms defining the dihedral rotation are taken at the relaxed molecular geometry and are kept constant when calculating the voltages for different dihedral angles. Similarly to the linear strain case, this degree of freedom allows us to manipulate the (binding) distances between carbonyl groups and a Mg atom.

The constrained calculations were performed with two reference structures to estimate the influence of initial conditions on the computed voltage changes. One structure represents the charged and the other the discharged state of the material. The discharged state is given by BipO2 with an attached Mg atom, i.e., Mg(C_12_H_8_O_2_)—referred to as MgBipO2. The charged state is given by BipO2, using the close-carbonyl group configuration because this is the configuration assumed by the molecule directly after the detachment of the Mg atom. The effect of Mg attachment, i.e., discharging, results in differences in the structure of the organic part of the molecule, which is most noticeable in the bond length and the dihedral angle between the phenyl-like rings. For the charged state (i.e., BipO2 without Mg) shown in Figure 4A, the bond length between the phenyl rings is 1.39 Å and has a dihedral angle of 30° while for the discharged system (MgBipO2) shown in Figure 4B, the bond length and dihedral angle increase to 1.51 Å and 71°, respectively. The impact on the computed properties with the different initial configurations in the constrained optimization is analyzed in Figures 4C–F. In these figures, we also compare the effect of keeping all atomic positions of the molecular structure fixed (i.e., only the position of the Mg atom is optimized—labeled as “frozen”) to the frozen dihedral angle calculation (i.e., all atomic positions but the ones defining the dihedral angle are allowed to relax during the structure optimization—labeled as “γ-frozen”).

Figure 4C compares the change of interaction energy (E_ad_) and Figure 4D shows the computed voltage vs. Mg^2+^/Mg for both of the reference structures, i.e., BipO2 and MgBipO2. The dihedral angle change of the configuration with strongest binding differs by around 60° between the BipO2 and MgBipO2 references. This is a consequence of the dihedral angle increase under Mg attachment.

For the MgBipO2 reference system, the binding energy between the Mg atom and the BipO2 molecule is −5.5 eV when fully relaxed (i.e., MgC_12_H_8_O_2_), corresponding to a voltage of 2.0 V vs. Mg^+2^/Mg. Under dihedral rotation, the binding energy reduces to −1.1 and −1.7 eV (an energy decrease of 4.4 eV and 3.8 eV) for the frozen and γ-frozen optimization leading to a voltage of −0.2 and 0.1 V vs. Mg^2+^/Mg (i.e., changes of 2.2 and 1.9 V), respectively. Both maxima are at a dihedral angle change of ca. 120°, which yields an almost flat geometry. The steric hindrance in the latter configuration is present in the molecular structure with and without the attached Mg atom. The binding energy weakening and voltage decrease is then a consequence of the removal of one of the Mg-O bonds. The loss of one bond (or reduction of bond order) also results in a reduced charge transfer. This is supported by the binding energy and voltage computed at Δγ of 180°. In this configuration, the carbonyl groups have a large distance from each other, and a Mg atom can only bind to one of them. The binding weakens by ca. 3 eV and the voltage takes a value of 0.8 V—values similar to the ones seen with the bond release mechanism in linear strain applied to PAD (see above) indicating that these values are somewhat characteristic of a single Mg-O bond in these molecules and the voltage reduction is caused by the loss of a Mg-O bond.

Similar changes but of smaller magnitude are seen in the BipO2 reference calculations. The curves are shifted by around 60°, which follows the change of the dihedral angle under Mg attachment during full optimization. The strongest interaction between the Mg atom and the BipO2 reference molecule yields a binding energy of −3.3 (−2.1) eV corresponding to a voltage of 0.9 (0.2) V vs. Mg^2+^/Mg for the γ-frozen (frozen) optimization. Dihedral rotation changes these values by 2.6 (1.8) eV setting the lower estimate of the voltage change to 1.3 (0.9) V. This is given at 60° for the frozen and γ-frozen BipO2 reference. The voltages reach their maximum because this configuration is most similar to the fully optimized structure of MgBipO2. Interestingly, the voltage curve is changing sign for both cases, frozen and γ-frozen constrained geometry calculations. In principle, this could indicate a polarity change of direct current. Then, the charging cycle could be replaced by a mechanical formation. However, the calculation misses to account for any aggregate state contribution to the voltage which is in the order of 1 V. This may shift the voltage curve above the point of polarity change. Nevertheless, the principle should be transferable to other materials. Depending on the onset of the voltage-capacity curve, the strain induced polarity change may be applicable to low-voltage electrode materials in general.

For the tested γ-frozen configurations, the LUMO shift of the molecule has a range of 0.63 eV and the HOMO shift of the MgBipO2 has a range of 0.4 eV. The frozen cases also have ranges of 0.6 eV or less. The Mulliken charges are between 0.9 and 0.5 *q* at 0 and 150° for MgBipO2 (frozen and γ-frozen) and 0.6 and 0.1 *q* for the BipO2 configuration at 30 and 150°, respectively. This means that the bond between Mg and the molecule becomes less ionic and weakens due to a dihedral rotation. In general, this can be seen in a reduction of bond order or the loss of a bond. In the context of the calculations, it is the loss of one of the Mg-O bonds in MgBipO2. Thus, structural effects are a major factor to be considered besides the observed small changes in electronic structure for dihedral angle controlled voltage changes in this molecule and potentially similar systems.

Figures 4E,F compare the effect of strain energy *E*_*S*_ when the Mg atom is removed and when it is present, respectively. Without an attached Mg atom (Figure 4E), the strain is minimized at round 120° for BipO2 and MgBipO2, as expected by the energetically preferred structure of BipO2 in which the carbonyl groups have a large separation distance. The transition potential of the rotation of BipO2 is ca. 0.9 and 1.4 eV for the γ-frozen and frozen calculation, respectively. For the MgBipO2 reference system, the molecule stores strain energy at the optimized structure due to the dihedral rotation under Mg attachment. Without Mg atom, this is partly released when the dihedral rotation reaches values similar to the one of the relaxed BipO2. In contrast, the strain energy increases by dihedral rotation when a Mg atom is attached. The max. values increase to more than 2 eV for all cases, see Figure 4F, and the increase in strain energy correlates roughly with a decrease in interaction strength with Mg in the case of the MgBipO2 reference. Since the distances between the redox-active groups and the Mg atom increase with increasing change of the dihedral angle, this correlation can be understood from the elongation and eventual loss of bonds between redox-active groups and the Mg atom.

**Figure 5:**
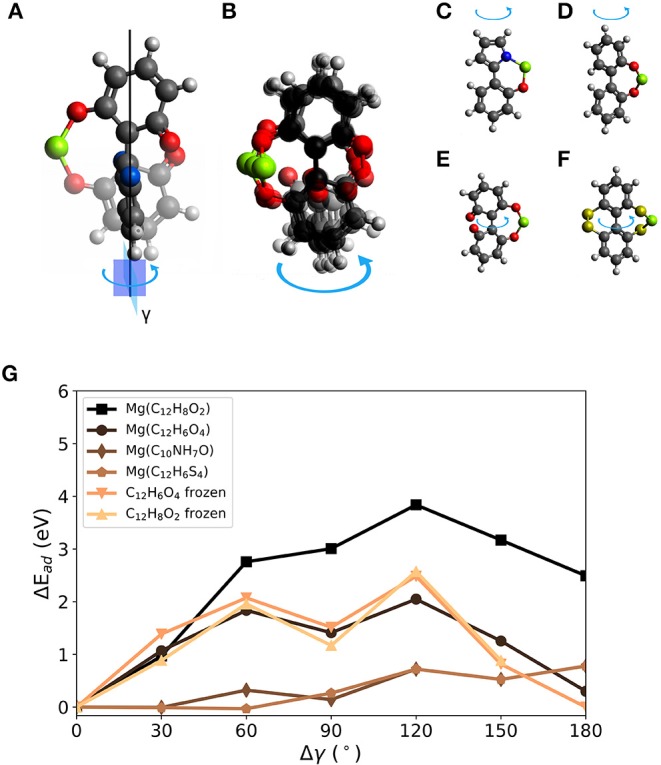
Rotation of the dihedral angle γ in biphenyl-derived molecules with carbonyl as redox-active group. **(A)** 1,1′-Bi(1,4-cyclohexadiene-1-yl) -2,2′,6,6′-tetrone (BCT) with chosen rotational axis (black vertical line) and angle (arrow). The initial structure is given with red O atoms and the final position with a changed dihedral angle between 6-membered rings indicated by blue O atoms on the ring. **(B)** Superposition of investigated rotated structures of BCT 0° to 180° in steps of 30°. Overview of molecules investigated for dihedral strain design: **(C)** C_10_NH_7_O, **(D)** C_12_H_8_O_2_ (BipO2), **(E)** BCT, and **(F)** C_12_H_6_S_4_. **(G)** Change of interaction strength with a Mg atom for different dihedral angles γ.

For BipO2, the voltage change by dihedral strain is estimated to be between 1.3 and 1.9 V. To further explore torsion on the molecular scale for voltage modulation, we proceed with three other molecules of a similar structure. Figure 5A shows a 1,1′-bi(1,4-cyclohexadiene-1-yl)-2,2′,6,6′-tetrone (BCT) molecule with a Mg atom attached in two configurations in which the dihedral angle between the 6-membered rings differ by 90°. We chose the molecules with a Mg atom attached as initial geometries. The blue arrow indicates the change of dihedral angle and the superimposed structures distinguish the atomic geometry before the rotation (faded out and with red oxygen atoms) and with 90° rotation (indicated by benzene-like ring with blue oxygen atoms). The loss of one Mg-bond and the bending of the molecular structure is visible. Figure 5B gives an overview of the structural changes after constrained relaxation for which the dihedral angle was changed in steps of 30° in a range from 0 to 180°. It is kept fixed during relaxation. The loss of one Mg-O bond for geometries with a dihedral rotation close to 90° is visible. Figures 5C–F show the investigated molecules. The systems were derived from the BipO2 molecule with a few modifications; this includes: **C**) combination of five- and six-membered ring–C_10_NH_7_O, **D**) and **E**) a different number of carbonyl groups on the phenyl-like rings and **F**) replacing carbonyl groups with R_2_C = S functional groups (BipS_4_). Figure 5G shows the changes in interaction strength (Δ*E*_*ad*_) as a function of change of dihedral angle γ for the structures shown in Figures 5C–F. As seen above, the change of interaction strength defines the change in voltage. However, a slight bend in the bond between the rings can cause differences in the interaction strengths at 0 and 180° due to a reduced structural symmetry.

In general, the tested molecules with carbonyl groups yield larger interaction strengths with Mg. Between BCT and BipO2, the differences are small. They have similar maximum voltages (e.g., 2.1 V for BCT). In contrast to BipO2, BCT and Mg interact via two carbonyl groups in each of the phenyl-like rings. At dihedral rotations of more than 90°, the distance between Mg and O atoms will increase again in BCT leading to significantly smaller Δ*E*_*ad*_ compared to BipO2. The other molecules have smaller maximum binding strengths. The interaction energies (voltages) of BipS4 and C_10_NH_7_O with Mg are −1.7 eV (0.1 V) and −3.1 eV (0.8 V), respectively.

For all tested molecules, the curves reach the point of maximal change of the interaction energy at a twisted structure with a dihedral angle changed by around 90 or 180°. This can be caused by the presence of one or two redox-active groups at the rotated ring or the bend structure due to Mg attachment on one side of a molecule. All molecules but C_10_NH_7_O significantly undergo a bending away from the attached Mg atom, in addition to the change of dihedral angle of the molecular structure. This can result in additional strain during the rotation. C_10_NH_7_O has a dihedral angle of 0° at full relaxation when Mg is attached. At a rotation of 180°, the distance between the redox-active groups and Mg is maximized and the interaction energy decreases by 0.8 eV. A similar decrease was observed for the C_12_H_6_S_4_ complex. For both complexes, the absolute decrease is smaller than for the MgBipO2 case since the binding strength is weaker. Although the C_12_H_6_S_4_ complex has two redox-active groups on each 6-membered ring, the maximal change of the interaction energy is not found at 90° but at 180° caused by a slightly bent structure and the changed interaction between S atoms. In all these cases, the strain can drastically decrease the interaction strength between molecule and Mg atom, and the interaction strength can be reduced by 80 % by torsion on the molecular scale.

Overall, the influence of the relative orientation of molecular planes and their redox-active groups, either within the same molecule or same complex but different molecules, can be a significant factor for changing the voltage-capacity curve if mechanical control on the molecular level can be achieved. In all tested cases, the interaction strength decreases between the molecules and the Mg atom leading to a lower theoretical voltage. While the molecular torsion does not increase the voltage, it could potentially be used to lower the charging voltage due to the weaker interaction strength. At certain dihedral angles, the binding strength can change by up to 2–3 eV translating to a voltage reduction of 1 to 1.5 V. Large steric hindrance can be expected that prevents a 180° rotation for practical cases.

### Design of Coordination Environment of Mg Cations

Mg atoms can bond in different ways to one or more redox-active groups of the active electrode material. They may also experience interaction with other molecules present in the electrode. Already in the example of the linear strain in the organic PAD-Mg complex shown above, the interaction strength with Mg differs when one or when two molecules simultaneously attach via a carbonyl group to Mg. Previously it was reported that the chemical environment can increase the voltage for organic Li and Na ion batteries, for instance, seen in the increase by ca. 1 V due to bulk phase (Manzhos, 2019 and references therein). Thus, it is not surprising if this also applies to organic electrode materials for MIB.

**Figure 6:**
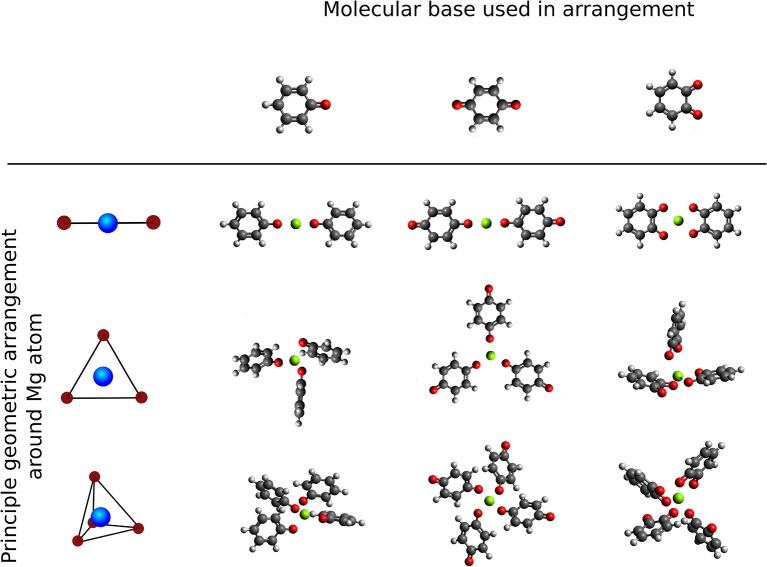
Overview of principle high symmetry arrangements around Mg atom centers (top to bottom: single molecule, linear, trigonal and tetrahedral) with different carbonyl based 6-membered ring molecules (left to right: principle geometries, phenyloxidanyl, benzoquinone and 1,2-Benzoquinone.

We study the effect of the local environment around the Mg atom created by molecules with carbonyl groups, that is the coordination of a different number and arrangement of carbonyl groups to a central Mg atom. The effect of a different number of redox centers in the same type of geometry was investigated by comparison between the benzoquinone (tBQE) and 1,2-benzoquinone (BQE). BQE and tBQE are structural isomers (C_6_H_4_O_2_) that allow us to probe the effect of the intramolecular carbonyl distance on the voltage. The effect of a different chemical character was tested by comparison between the tBQE and phenyloxidanyl molecule (C_6_H_5_O). Three different types of geometries were selected to test the influence of the arrangements, that are: linear, trigonal and tetragonal, displayed in Figure 6. It should be noted that in most cases, molecular systems without a central Mg atom show weak or no binding in our calculations, partly because we do not include van-der-Walls interactions (on purpose, as we are interested in electronic effects). Furthermore, we adapt Equation (2) by substitution $E_{r}^{mol}$ with $w\;E_{r}^{mol}$ where *w* is the number of molecules in the final geometry.

The computed interaction energies and resulting theoretical voltages for the fully relaxed systems (structures shown in Figure 6) are presented in Table 1. tBQE has voltages of 0.2 to 1.8 V when more than one molecule coordinates to the Mg cation while a single BQE molecule results in a formally negative voltage of −0.5 V. Phenyloxidanyl has a voltage of ca. 1.7–2.8 V that is higher than those of tBQE in comparable configurations possibly due to its radical character. The BQE based configurations result in even higher voltages (0.3 V for one molecule and 2.2–2.8 V for linear to tetragonal configurations) than for tBQE and are of similar magnitude as for phenyloxidanyl. Although BQE does not have a radical character, the increase in voltage compared to BQE could arise through the interaction of the positive Mg cation and the two negatively charged carbonyl groups of each BQE. A possible consequence for the design of active Mg cathode material is to create a chemical environment for the Mg atom with a large number of redox-active groups. The results show that the type of coordination geometry can lead to a, e.g., max. voltage of 2.8 V for BQE that is an increase of 2.7 V compared to a single molecule. The number of redox centers that coordinate to Mg is a key factor to achieve this high voltage and it can change the voltage by more than 2 V. Interestingly, chemical locality design can change a non-working electrode, e.g., single molecule tBQE, into a working electrode, e.g., a trigonal complex of tBQE.

**Table 1:** Formation energies (eV) [theoretical voltages (V) vs. Mg2+/Mg] for systems shown in Figure 6.

	**Phenyloxidanyl**	**Benzoquinone (tBQE)**	**1,2-Benzoquinone (BQE)**
Single molecule + Mg	−1.72 [0.10]	−0.44 [−0.53]	−2.11 [0.30]
Linear complex	−4.88 [1.68]	−2.01 [0.24]	−5.83 [2.16]
Trigonal complex	−6.44 [2.46]	−3.92 [1.21]	−6.98 [2.73]
Tetrahedral complex	−7.04 [2.77]	−5.13 [1.81]	−7.11 [2.80]

### A Promising Molecular Design

**Figure 7:**
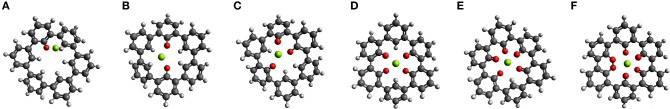
Optimized structures of MgC_36_O_n_H_24−n_ *n* = {1,2,3…6} where *n* = 1 **(A)** increases to *n* = 6 **(F)**.

The idea of combining structural and electronic design is applied to a complex ring system, a cycloparaphenylene molecule consisting of three fused biphenyl-like units (C_36_H_24_), shown in Figure 7. This molecular system combines the previously described effects of dihedral rotation, chemical environment, and it is possible to introduce electronic modifications, for example, by functionalization. We discuss possible modifications to an inner (with six H atoms, not shown) and an outer ring (with 18 H atoms). The bonds between the phenyl rings allow rather easy partial rotational changes. Up to six carbonyl groups can be in the inner ring. Then, the O atoms can take, e.g., a linear, trigonal, square-planar, tetragonal and bipyramidal-like symmetry for a central Mg ion inserted into the center of the molecule. Regarding the relative positions of the O atoms, these geometries are similar to the one shown in the section Design of Coordination Environment of Mg Cations. Of course, different combinations of sites of the carbonyl group are possible for two to four O atoms in the inner ring. The optimized geometries of the ring molecule with one to six carbonyl groups and an inserted Mg atom are shown in Figures 7A–F, respectively.

**Figure 8:**
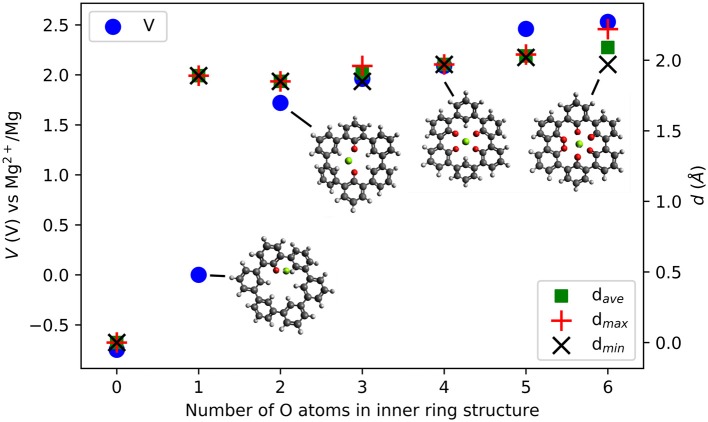
Computed voltage (V) vs. Mg^2+^/Mg (left axis) and Mg-O distances d {average, maximal, minimal} (Å) (right axis) in triple-biphenyl ring structures as a function of the number of O atoms in the inner ring of the molecule.

Figure 8 shows the voltage vs. Mg^2+^/Mg computed for the insertion of one Mg atom in the inner ring of the C_36_H_24_ molecule and its derivatives with different numbers of O atoms. There is no strong interaction between Mg atoms and the C_36_H_24_ molecule (no O atoms), resulting in a theoretical voltage of −0.75 V, i.e., the molecule is not suitable for Mg storage. The first carbonyl group increases the interaction strength and results in a slightly positive voltage. The most significant increase in the voltage is observed when increasing the number of inner carbonyl groups from one to two. This is similar to the cases of individual molecules with their carbonyl groups around the Mg atom. Introduction of more inner ring carbonyl groups increases the voltage to a value of slightly more than 2.53 V vs. Mg^2+^/Mg. Depending on the number of carbonyl groups, the structure of the molecule exhibits different dihedral angle rotations between six-membered rings when the Mg atom is inserted (the strain energy is estimated at 2.7 eV). In contrast, the presence of carbonyl groups without an inserted Mg atom has a minor effect on these angles.

Introducing carbonyl groups in the inner ring can serve two purposes. First, it introduces redox-active groups. Choosing O atoms is obvious since carbonyl groups are known to result in working organic electrode materials, e.g., BQE. Second, the O atoms have a larger electronegativity than H atoms, thus they have an electron-withdrawing effect and they can stabilize the LUMO energies in organic compounds. From zero to six O atoms, the LUMO decreases from −1.43 to −3.81 eV, a change of −2.38 eV. As a result, the electronic effect on the voltage increase is at most 1.19 V while the computed voltage change is 3.28 V. This indicates that also for this system, electronic effects alone cannot fully explain the voltage increase and the number and character of bonds formed between the Mg atom and the carbonyl groups must be considered.

**Figure 9:**
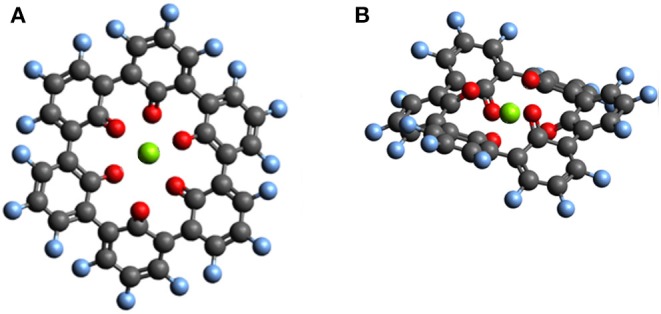
Top view **(A)** and side view **(B)** of the optimized structure of hypothetical C_36_F_18_MgO_6_ molecule for which a voltage of 3.51 V vs. Mg^2+^/Mg was computed.

**Figure 10:**
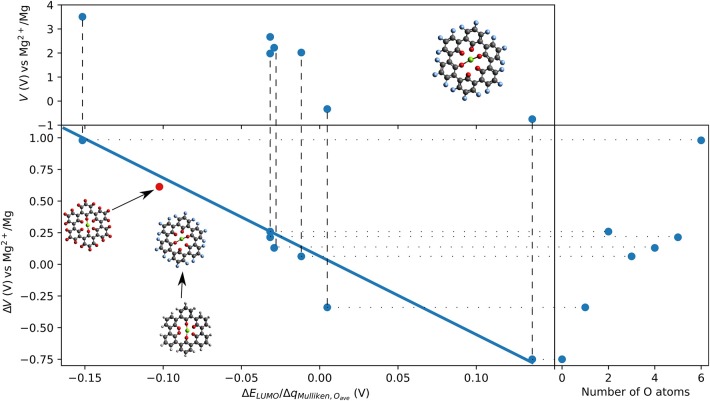
F-functionalization induced changes in voltage vs. Mg^2+^/Mg as a function of the ration of change of LUMO energy and Mulliken charges on O atoms in the inner ring of triple-biphenylene molecule. The insert shows a linear regression (solid blue line). One data point (red dot) shows the result of C_36_O_24_.

Outer ring functionalization gives another example of the interplay between electronic and structural effects on the voltage. We explore functionalization with F and with O atoms, and the resulting effect on the voltage for the insertion of one Mg atom. In this way, the contribution from the chemical locality (inner ring structure) and electronic changes through functionalization can be combined. The former option (substitution with F atoms leading to C_36_F_18_O_6_, structure is shown in Figure 9) can increase the theoretical voltage to 3.51 V vs. Mg^2+^/Mg (an increase by 1 V) at full outer ring fluorination and the latter option (O atoms–C_36_O_24_) results in a maximal voltage of 3.17 V (an increase by 0.64 V). An overview of voltages and voltage changes between the F-functionalized and the non-functionalized ring structure is shown in Figure 10, top and bottom panel, respectively. The right panel of Figure 10 shows the voltage changes depending on the number of carbonyl groups in the inner-ring. The LUMO of the F functionalized molecule is stabilized by 1.46 eV and the O functionalized one is stabilized by 2.9 eV. The counter-intuitive relation between an increase in voltage and LUMO stabilization requires to extend a model that is only based on LUMO energy changes to understand the atomistic reasons behind the observed effects.

While the change of LUMO energy can often explain any voltage increase by electronic effects, we introduce a contribution accounting for the chemical environment to extend the LUMO based empirical model. Since F atoms have a larger electronegativity than O and H atoms, a charge redistribution can take place when the molecule is functionalized with F atoms. To consider this in a simple approach, we measure the charge redistribution on the redox-active atoms (here the O atoms in the inner ring). Since we are only interested in changes due to functionalization of the outer ring, the charge redistribution can be estimated by the Mulliken charges.

We provide a simple model based on the LUMO energy changes and the changes in the Mulliken charges of the redox-active atoms (nearest neighbor to inserted Mg atom) to estimate a voltage increase for Mg batteries. The model is based on the assumption that the energy level of the LUMO and the Coulombic charges estimated as point charges surrounding a central Mg ion will contribute to the interaction strength. Furthermore, we assume that the change of the voltage is proportional to the change of LUMO energy, and that it is indirectly proportional to the averaged change of charge on the redox-active centers due to functionalization.

The linear regression (shown as solid blue line in the bottom panel of Figure 10) for organic Mg electrode materials operating by reduction confirms a relatively linear trend over the ratio of change in LUMO eigenvalue and change in Mulliken charges on the redox-active groups that are involved in the redox process. The suggested model can also explain our previous observation that electronic modification in the coronene molecule does not result in increased voltages for Mg batteries while it affects the interaction with Li and Na ions (Lüder et al., 2017b). Furthermore, the model was confirmed with the result of the O-functionated ring structure that is indicated as red dot in Figure 10 and it may find application in other systems, too.

Among all investigated structures, the C_36_O_24_ molecule might be the most promising one for further investigation aiming to optimize its potential vs. Mg^2+^/Mg. It has a conceptual similarity to oxocarbon salts (Zhao et al., 2016) which showed promise for monovalent batteries. For this system, we computed multiple Mg atom attachments since the O functionalization introduced more carbonyl groups that can be used to store more Mg atoms. The capacity corresponding to the attachment of up to 5 Mg atoms results still in voltages of ca. 2 V or more vs. Mg^2+^/Mg. As the attachment takes place at the outer ring carbonyl groups, these voltages are a lower estimate and chemical locality of bulk materials constituted by neighboring molecules could further increase the voltage at higher capacities.

## Conclusions

Organic Mg ion batteries are still in their early stages of development. Specifically, it is challenging to design insertion type active high-voltage electrode materials; many types of molecules that show good performance in monovalent batteries do not work for Mg ion batteries and do not bind Mg. Even for molecular materials with appreciable interaction energy with Mg, bivalency means very low voltages. In this work, we have explored computationally design strategies that could be used to increase voltages, applying them to several prototypical molecular systems. We considered electronic structure design, structural changes/functionalizations, strain design as well as the design of Mg coordination environment.

We find that it is possible to increase the voltage of organic electrodes for Mg batteries by several volts by a combination of electronic and structural design. Electronic structure design based on LUMO stabilization, however, shows severe limitations to voltages of not more than 2 V for most materials. In particular, we demonstrate that an increase of voltage by 4.3 V to a maximal voltage of 3.5 V vs. Mg^2+^/Mg can be achieved when electronic effects are combined with engineering of the local chemical environment. Designing coordination environment for Mg atoms can change the voltage by 2.0 to 2.5 V already for small molecules and appears to be a promising strategy.

Three types of strain that are linear strain, bending and dihedral strain can influence the interaction strength to Mg; here, we found they lead to lower interaction energies/voltages. Strain design may find potential application in lowering the charging potential if mechanical control at the molecular level is achievable. A reduction of 0.5 V for bending and 1–2 V for linear and dihedral strain may be achievable.

Based on the results obtained with the ring structures that combine electronic effects with effects of the chemical locality, we provide an empirical model based on two computable molecular properties, LUMO energy change and changes of Mulliken charges, to estimate the voltage change due to structural modifications such as fluorination.

In this paper, we explored what would be theoretical limits of voltage increase by modifications of molecular and electronic structure. Some of these modifications could be achieved in a way which is intrinsic to a single molecule (e.g., doping, use of functional groups causing steric hindrance to induce strain, or coordination to several redox-active centers of the same molecule) and is expected to be stable under battery cycling. Other modifications that we explored (e.g., linear or dihedral strain or coordination to redox-active centers of several molecules) would require development of practical design strategies to stabilize these environments (e.g., anchoring (Chen and Manzhos, 2015) to approach the theoretical limits delimited here.

## Data Availability Statement

All datasets generated for this study are included in the article/Supplementary Material.

## Author Contributions

JL: calculations, data analysis, and text. SM: conceptualization, data analysis, and text.

### Conflict of Interest

The authors declare that the research was conducted in the absence of any commercial or financial relationships that could be construed as a potential conflict of interest.
