# Supplementary material for: First-Principle Insights Into Molecular Design for High-Voltage Organic Electrode Materials for Mg Based Batteries
Source: Front Chem. 2020 Feb 18;8:83. doi: 10.3389/fchem.2020.00083 (PMC7045799; doi:10.3389/fchem.2020.00083)
Supplement: Supplementary file 1 [file Data_Sheet_1.PDF]

Supporting information for:

First-principle insights into molecular design for high-voltage organic electrode materials for Mg based batteries

Johann Lüder[1*] and Sergei Manzhos[2*]

[1] Department of Materials and Optoelectronic Science, National Sun Yat-Sen University, Kaohsiung, Taiwan

[2] Centre Énergie Matériaux Télécommunications, Institut National de la Recherche Scientifique, 1650, boulevard Lionel-Boulet, Varennes QC J3X1S2 Canada

*** Correspondence:**
Corresponding Author

johann.lueder@mail.nsysu.edu.tw
sergei.manzhos@emt.inrs.ca

The analysis of the electronic effects in the main article is based on a dataset of small organic molecules with a ring structure. The molecules were functionalized with, e.g., F atom and carbonyl groups to change their redox behavior and their electronic structure. For each molecule, electronic properties were computed such as the HOMO and LUMO eigenvalues, as well as the theoretical voltage vs Mg^{2+}/Mg for an attachment of one Mg atom. These results were used to group the molecules according to the outcome of the k-means algorithm described in the main article.

Figure S1 and S2 show the structures of molecules labeled as 'Negative V vs Mg^{2+}/Mg' in the main text, that is the green area and green data points in Figure 1 of the main article. Most of these molecules have weak interaction with Mg atoms. Figure S3 and S4 show the structures of molecules with a low LUMO energy (labeled as 'Deep LUMO materials'), that is the blue area and blue data points in Figure 1 of the main article. Figure S5 shows the molecular structure of the high LUMO materials indicated by the purple area and purple data points in Figure 1a and 1b of the main article.

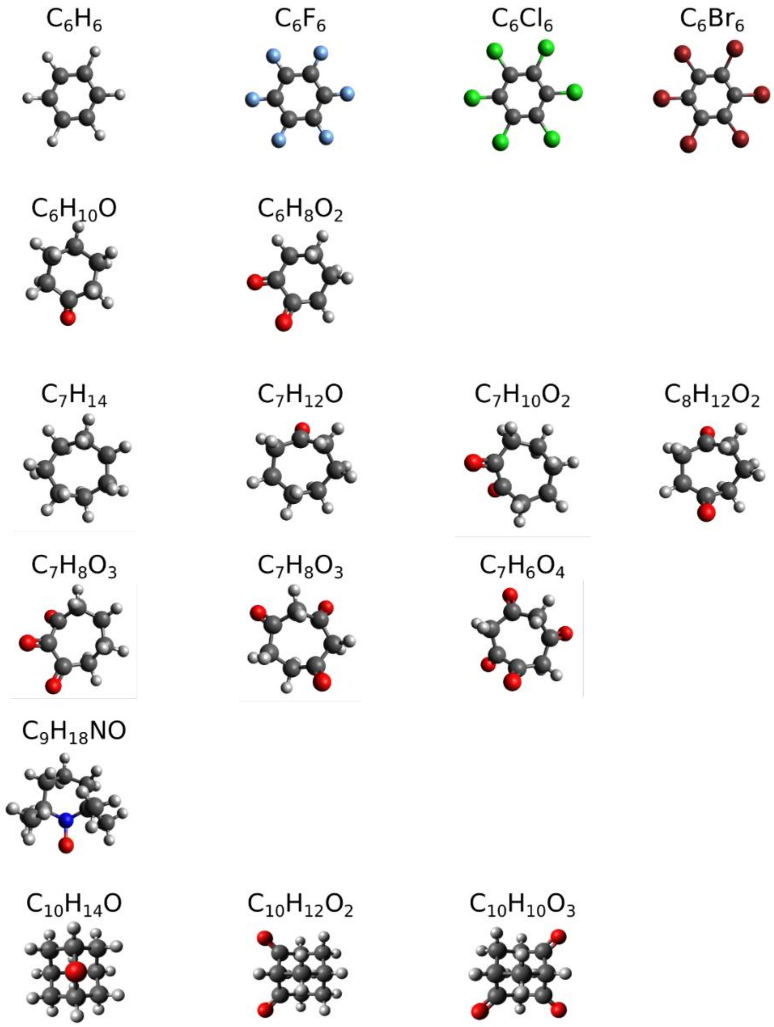

Figure S1. Part I of the molecules of the negative V (i.e. non-working) electrode materials cluster determined by a k-means algorithm. The color code is carbon (gray), oxygen (red), hydrogen (white), fluorine (light blue), chlorine (light green) and bromine (drak red) here and below.

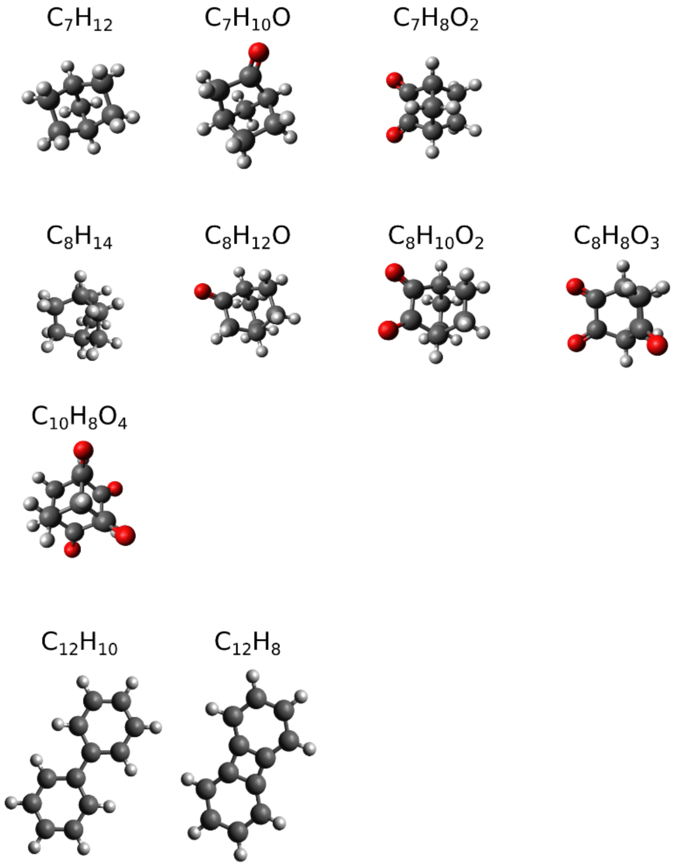

Figure S2. Part II of the molecules of the negative V (i.e. non-working) electrode materials cluster determined by a k-means algorithm.

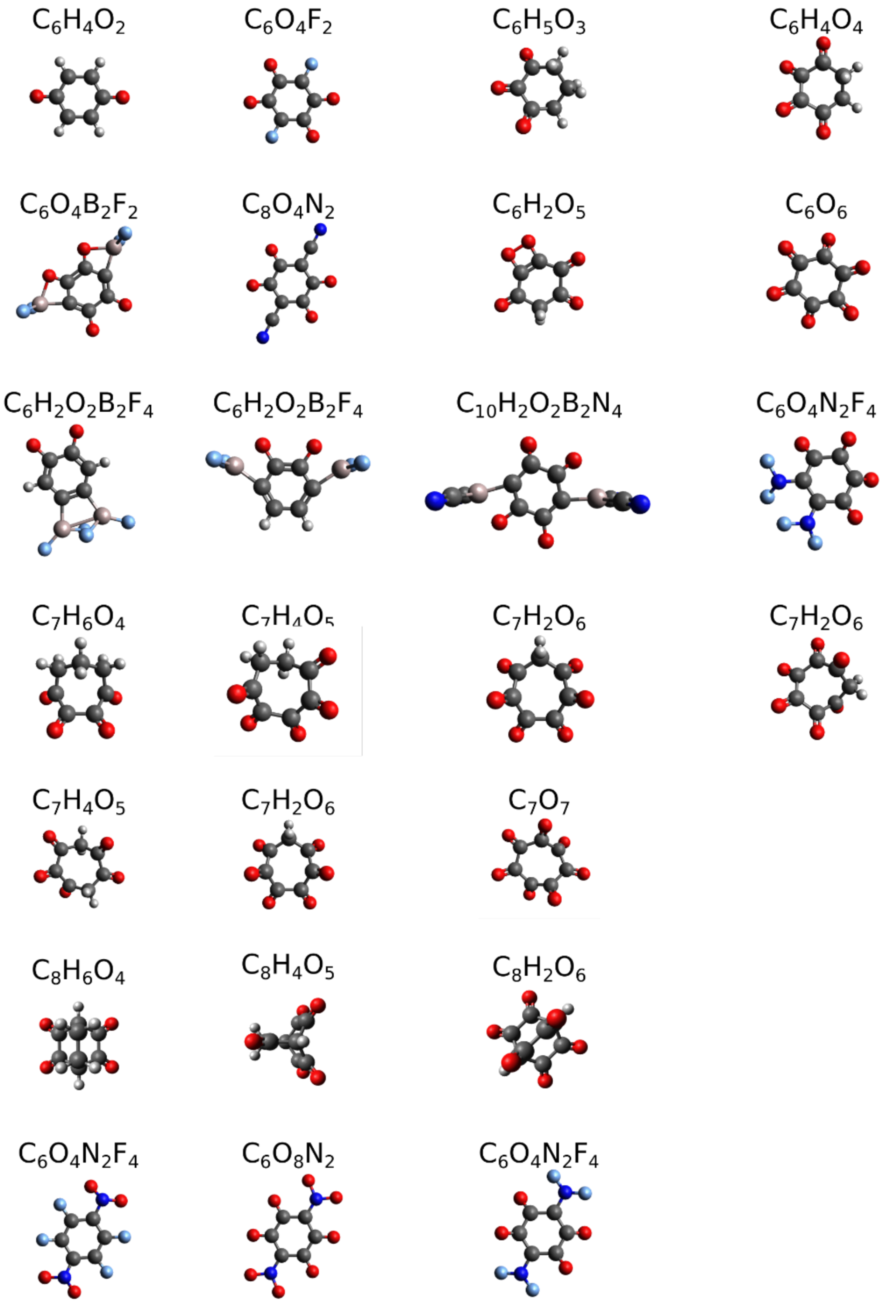

Figure S3. Part I of the molecules of the deep LUMO materials determined by a k-means algorithm. Color code is nitrogen (blue) and boron (pink) here and below.

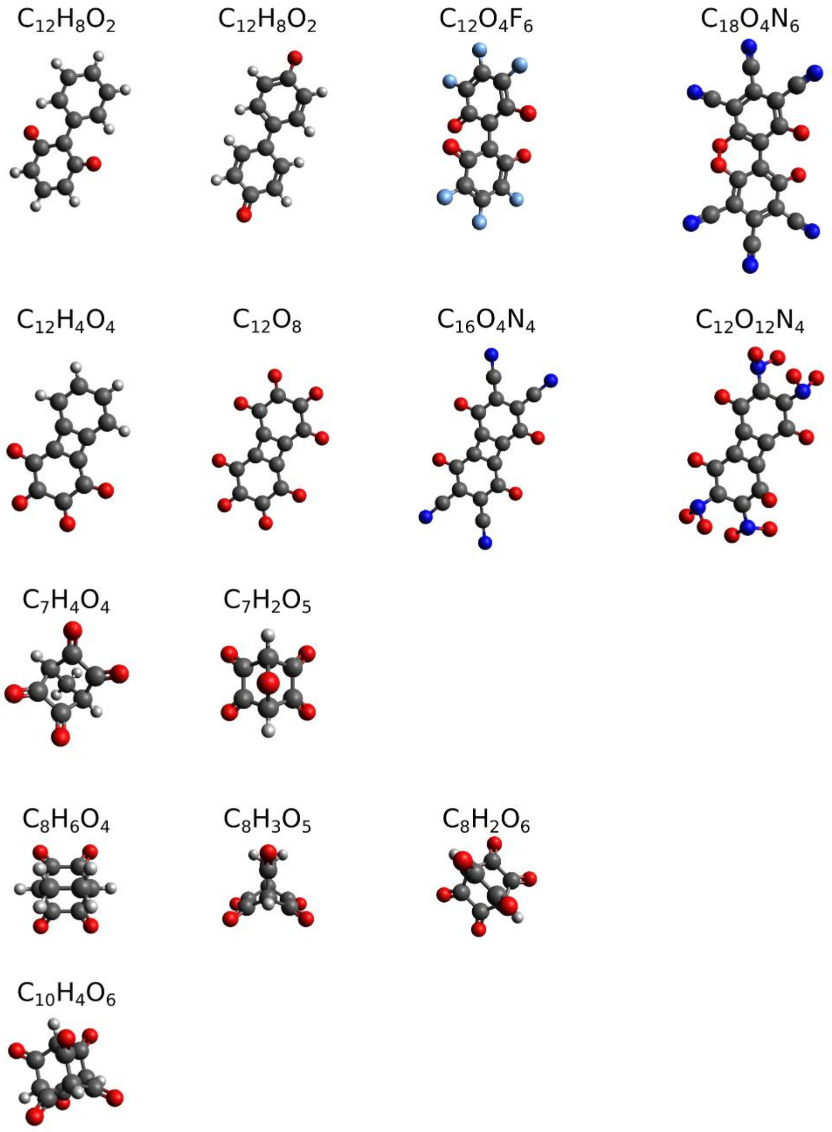

Figure S4. Part II of the molecules of the deep LUMO materials cluster determined by a k-means algorithm.

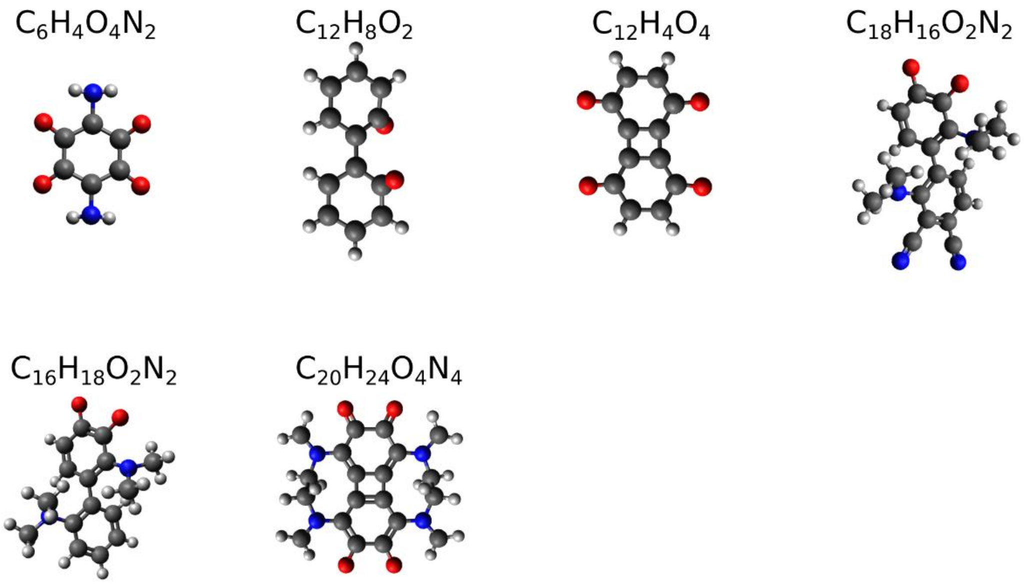

Figure S5. Molecules of the high LUMO materials cluster determined by a k-means algorithm.
